# Aberrant RNA Splicing in Cancer and Drug Resistance

**DOI:** 10.3390/cancers10110458

**Published:** 2018-11-20

**Authors:** Bi-Dar Wang, Norman H. Lee

**Affiliations:** 1 Department of Pharmaceutical Sciences, School of Pharmacy, University of Maryland Eastern Shore, Princess Anne, MD 21853, USA; 2 Department of Pharmacology and Physiology, School of Medicine and Health Sciences, George Washington University, GW Cancer Center, Washington, DC 20037, USA

**Keywords:** alternative splicing, spliceosome, splicing factor, drug resistance, splice switching oligonucleotide, splicing factor inhibitor

## Abstract

More than 95% of the 20,000 to 25,000 transcribed human genes undergo alternative RNA splicing, which increases the diversity of the proteome. Isoforms derived from the same gene can have distinct and, in some cases, opposing functions. Accumulating evidence suggests that aberrant RNA splicing is a common and driving event in cancer development and progression. Moreover, aberrant splicing events conferring drug/therapy resistance in cancer is far more common than previously envisioned. In this review, aberrant splicing events in cancer-associated genes, namely *BCL2L1*, *FAS*, *HRAS*, *CD44*, *Cyclin D1*, *CASP2*, *TMPRSS2-ERG*, *FGFR2*, *VEGF*, *AR* and *KLF6*, will be discussed. Also highlighted are the functional consequences of aberrant splice variants (*BCR-Abl35INS*, *BIM-γ*, *IK6*, *p61 BRAF V600E*, *CD19-∆2*, *AR-V7* and *PIK3CD-S*) in promoting resistance to cancer targeted therapy or immunotherapy. To overcome drug resistance, we discuss opportunities for developing novel strategies to specifically target the aberrant splice variants or splicing machinery that generates the splice variants. Therapeutic approaches include the development of splice variant-specific siRNAs, splice switching antisense oligonucleotides, and small molecule inhibitors targeting splicing factors, splicing factor kinases or the aberrant oncogenic protein isoforms.

## 1. Alternative Splicing

Alternative splicing (AS) is a post-transcriptional process leading to the generation of alternative mRNA transcripts that encode structurally and perhaps functionally different protein isoforms. Genome-wide analysis of the human transcriptome based on expressed sequence tags (ESTs) have identified a diverse repertoire of splice variants [1]. ‘Splicing-sensitive’ microarray and next-generation deep sequencing results suggest that more than 95% of human genes are transcribed into pre-mRNAs that undergo AS [2,3,4,5], which increases the complexity of human transcriptome and explains why ~20,000 protein-coding genes in the human genome can give rise to more than 250,000 proteins in the human proteome. On average, a human gene contains 8 to 10 coding exons of variable length separated by non-coding introns that can be 10 to 100 times longer [6]. Following transcription of intron-containing genes, the pre-mRNA undergoes maturation where the spliceosome machinery catalyzes the excision of introns and ligates exons together. The process of AS occurs when different exons are retained or excluded to generate alternative mRNA transcripts, which allows a single gene to produce multiple mRNA variants, resulting in protein isoforms that can have distinct structural and drastically different biological activities [7]. Consequently, AS dramatically expands the protein-coding repertoire of higher eukaryotes. The expression of specific variants is regulated in a developmentally and tissue-specific manner [4,8,9,10,11,12,13,14]. Moreover, coordinated regulation of AS within gene networks plays a key role in cell differentiation and specialization [15,16,17].

**Figure 1:**
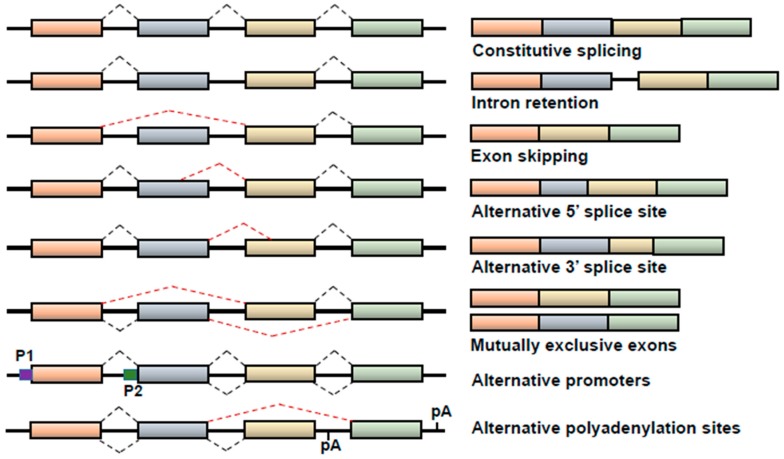
Different modes of alternative RNA splicing. Different modes of alternative splicing (AS), including constitutive splicing, intron retention, exon skipping, alternative splice site selection (5′ and 3′), mutually exclusive splicing, alternative promoter selection and alternative polyadenylation sites [19,20]. The pre-mRNAs are shown on the left panel, and the mature mRNA variants following AS are shown on the right panel.

There are several different mechanistic modes of AS in human cells. The different splicing events include: (1) Intron retention—the intron is retained in the mature mRNA transcript; (2) exon skipping—the entire exon is spliced out of the mature mRNA transcript; (3) alternative 5′- or 3′-splice sites—alternative selection of 5′- or 3′-splice sites that leads to the retention of a smaller exon; (4) mutually exclusive exons—different exon combinations are selected/retained to generate different splice variants; (5) alternative promoter selection—different polymerase II promoters are selected/occupied to generate alternative 5′-ends (it should be noted that this particular event is transcription factor-mediated and not splicing factor-mediated) [18]; and (6) alternative polyadenylation sites—selection of different polyadenylation sites to generate alternative 3′-ends (Figure 1). In-frame splicing events can produce mRNA variants that encode structurally and perhaps functionally different protein isoforms, contributing to the development and/or progression of cancer and/or treatment response.

## 2. Aberrant Alternative Splicing in Cancer

Cancer cells evolve by developing mechanisms that allow adaptation to their microenvironment. The cellular plasticity offered by AS enables cancer cells to produce protein isoforms favoring tumor growth and/or spreading [21]. Genome-wide approaches have revealed that large-scale AS occurs during tumorigenesis [22], and the genomic portraits of AS patterns have proven useful in the classification of tumors [23,24,25]. Reports of aberrant splicing events and alterations in ratios of alternatively spliced transcripts in different cancers have been noted, including breast, colon, prostate, lung, ovarian, brain and pancreatic cancers [20,25,26,27,28,29]. These events result in novel transcripts not observed in normal cell counterparts. It has been reported that nearly all areas of tumor biology are affected by AS, including metabolism, apoptosis, cell cycle control, invasion, metastasis and angiogenesis [23,30].

**Figure 2:**
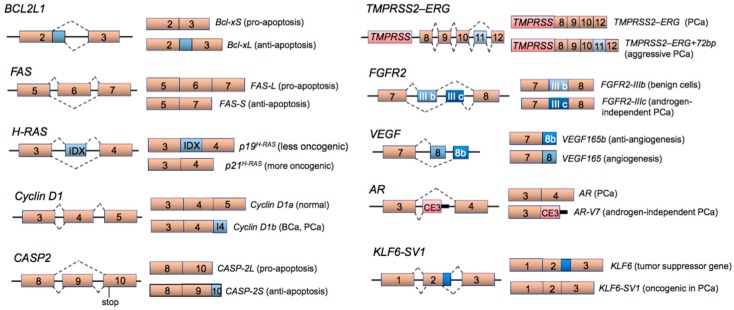
Alternative splicing events in cancers. Examples of aberrant splice variants and oncogenic consequences for *Bcl-xL*, *FAS-S*, *p21H-RAS*, *Cyclin D1b*, *CASP-2S*, *TMPRSS2-ERG+72bp*, *FGFR2-IIIc*, *VEGF-165*, *AR-V7* and *KLF6-SV1* [20,43,47]. Abbreviations: *TMPRSS2–ERG+72bp*, TMPRSS2–ERG fusion transcript with inclusion of a 72-bp exon [48]; BCa, breast cancer; PCa, prostate cancer; CE3, cryptic exon 3. Overexpression of splice variant *TMPRSS2–ERG+72bp* has been correlated to aggressive prostate cancer with poor clinical outcome [49]. The pre-mRNAs are shown on the left panel, and the mature mRNA variants (only the exons surrounding the differential splicing event are shown) following AS are shown on the right panel.

One of the earliest examples of alternative spliced variants with opposing apoptotic effects is the B-cell lymphoma 2-like 1 (*BCL2L1*) gene (Figure 2). The *BCL2L1* pre-mRNA can be alternatively spliced to encode two protein isoforms, anti-apoptotic *Bcl-xL* (long isoform) and pro-apoptotic *Bcl-xS* (short isoform) [31,32]. High *Bcl-xL*/*Bcl-xS* mRNA ratios, associated with greater tumor cell survival, can be found in a number of cancer types, including human lymphoma, breast cancer, prostate cancer and human hepatocellular carcinoma [33,34,35,36]. Antisense oligonucleotides can effectively modulate (i.e., decrease) the *Bcl-xL*/*Bcl-xS* ratio to favor prostate cancer cell sensitization to radiation and chemotherapeutic agents [36]. Another example of an apoptosis-related gene that undergoes alternative RNA splicing in cancer cells is the *FAS* receptor gene. Expressed on the cell surface of many cell types, the *FAS* receptor is activated by the *FAS* ligand produced by cytotoxic T cells, which initiates a death-signaling cascade leading to apoptosis [37]. There are at least three short mRNA variants of *FAS* missing the encoded transmembrane domain and the resulting translated protein isoforms are presumably secreted by cancer cells and act as decoy receptors for the *FAS* ligand, thus allowing cancer cells to escape from apoptosis [38,39]. Caspase-2 (CASP-2) is activated by different stimuli, such as reactive oxygen species (ROS) [40,41], death receptor ligands, heat shock [42] or cytotoxic drugs. The *CASP-2* gene produces multiple mRNA splice variants, including variants with pro-apoptotic or anti-apoptotic properties. *CASP-2L* encodes a full-length caspase-2 protein, which is expressed in most tissues and promotes apoptosis. However, splice variant *CASP-2S*, resulting from an exon 9 retention event that leads to a premature termination, encodes a truncated protein lacking the active domain and thereby inhibits cell apoptosis [43]. Previous studies have shown that *CASP-2S* overexpression promotes anti-apoptotic activities and protects cells from cytotoxicity by chemotherapeutic agents, such as etoposide in leukemic/lymphoma cells [44,45,46].

AS also plays a role in promoting proliferative, invasive and/or metastatic behavior in cancers. An AS event involving the *HRAS* oncogene results in the exclusion of a previously uncharacterized exon (named intron D exon or IDX) due to an intronic mutation in *HRAS* [50]. The IDX-containing *HRAS* mRNA variant encodes a truncated P19 protein that is less oncogenic compared to the *HRAS* variant missing IDX, which encodes a full length P21 protein. Cyclin D1 functions as a regulator in cell cycle progression, and its association with cyclin-dependent kinase 4/6 (forming CDK4/6/cyclin D1 complex) promotes the phosphorylation of tumor suppressor retinoblastoma protein (RB), leading to de-repression of the E2F transcription factor and subsequent enhanced cell proliferation [51,52]. Overexpression of cyclin D1 results in upregulation of CDK4/6 activity and contributes to neoplastic growth [53,54]. It has been shown that a common polymorphism in the cyclin D1 (*CCND1*) gene is associated with the generation of an alternative transcript, termed cyclin D1b. Compared to the full-length canonical mRNA variant cyclin D1a that retains all five exons, the cyclin D1b variant contains four exons and intron 4 due to a G/A870 polymorphism at the exon 4-intron 4 boundary. The latter variant cyclin D1b is more tumorigenic [55], associated with poor prognosis in ER-negative breast cancer [56], and more resistance to antiestrogen therapy in ER-positive breast cancer [57]. *CD44* was among the first genes with splice variants specifically associated with metastasis, where variants containing exons 4–7 (v4–7) and 6–7 (v6–7) were shown to be expressed in a metastasizing pancreatic carcinoma cell line, but not in the corresponding primary tumor [58]. The fibroblast growth factor (FGF) family and their transmembrane receptors (FGFRs) are thought to be of importance in prostate carcinogenesis. AS of *FGFR2*, resulting in the switching of the *FGFR2-IIIb* variant to the *FGFR2-IIIc* variant, is associated with malignant transformation and androgen insensitivity [59]. Moreover, high affinity binding between the FGFR2-IIIc isoform and its ligand FGF8b is significantly associated with higher Gleason grade and clinical stage of prostate cancer [60]. Vascular endothelial growth factor (VEGF) is up-regulated in solid tumors and is associated with angiogenesis. Typical splicing of *VEGF* results in an anti-angiogenic splice variant *VEGF165b*, which is widely expressed in normal cells and tissues, but is down-regulated in prostate cancer [61] and potentially serves as an anti-angiogenic, anticancer therapeutic via either controlling *VEGF* splicing or targeted delivery [62]. Kruppel-like factor 6 (*KLF6*) is a tumor suppressor gene, and AS of *KLF6* results in a dominant-negative splice form *KLF6-SV1* (splice variant 1), which plays a critical role in promoting cell proliferation, survival, migration and angiogenesis of prostate cancer [63,64]. Moreover, *KLF6-SV1* is overexpressed in metastatic prostate cancer and associated with the increased metastasis and poor clinical outcome [65].

Aberrant splicing can also lead to splice variants encoding protein isoforms that impact distinct signaling pathways. Cyclin D-binding Myb-like Protein 1 (*DMP1*) is a critical tumor suppressor gene in breast cancer, and *DMP1* splicing results in splice variants *DMP1α*, *DMP1β* and *DMP1γ* [66]. DMP1α is tumor suppressive by transcriptionally up-regulating ARF, leading to apoptosis and anti-tumorigenesis in a P53-dependent manner [67]. In contrast, DMP1β has pro-proliferative oncogenic activity that is P53-independent [67]. Overexpression of *DMP1β* mRNA has been observed in 60% of breast cancers [66] and is associated with the poor clinical outcome [66,67]. Another example is *CXCR3*, a gene involved in cancer metastasis and inflammatory diseases [68,69]. *CXCR3* splice variants, *CXCR3A*, *CXCR3B* and *CXCR3Alt*, encode protein isoforms exhibiting differential activation of Gαi, β-arrestin and ERK1/2 in response to cytokine stimulation. CXCR3A fully activates Gαi and ERK1/2 and partially stimulates β–arrestin recruitment in response to CXCL10. CXCR3B induces β–arrestin recruitment but weakly activates ERK1/2 in response to CXCL11. Lastly, CXCR3Alt fails to activate Gαi, β-arrestin and ERK1/2 in response to its cytokine ligands [70].

## 3. Splicing Factors in Cancer Development and Progression

### 3.1. Aberrant Expression of Splicing Factors

Alternative RNA splicing is regulated/catalyzed by a large and highly dynamic protein complex called the spliceosome [71,72]. The spliceosome complex is composed of five small nuclear ribonucleic acids (snRNA U1, U2, U5, U5 and U6) and about 200 protein components [71,73]. Among the associated 200 proteins, there are two well-studied RNA splicing factor families, the serine-arginine rich splicing factors (SRSF) and the heterogeneous nuclear ribonucleoprotein (HNRNP) proteins [71,73]. Mechanistically, SRSF proteins bind to exon splicing enhancers (ESEs) and intronic splicing enhancers (ISEs), while HNRNP proteins tend to bind exonic splicing silencers (ESSs) and intronic splicing silencers (ISSs). Therefore, SRSF and HNRNP proteins have been implicated in promoting exon inclusion and exon skipping during the process of alternative mRNA splicing [74,75,76,77].

Incorrect assembly of the spliceosome complex due to aberrant expression and/or mutations of spliceosome components often leads to splicing abnormalities. Mis-regulation of splicing factors has been recently identified as a mechanism underlying aberrant mRNA splicing programs associated with cancer development and progression. Several SRSF and HNRNP proteins are found up-regulated in human cancers [78]. SRSF1 (also known as SF2/ASF) is the first splicing factor to be identified as a proto-oncogene in human cancers [79]. Previous studies revealed that SRSF1 is up-regulated in different types of human cancers, including colon, breast, thyroid, small intestine, kidney and lung cancers [79,80]. Overexpressed SRSF1 has been shown to be involved in aberrant AS of *RON*, *BIN1*, *MNK2*, *S6K1*, *BCL2L1* and *MCL1* pre-mRNAs, which results in enhanced expression of oncogenic protein isoforms of RON [79], MNK2 and S6K1 [80], enhanced expression of anti-apoptotic isoforms Bcl-xL and MCL-1L [81], and loss of the tumor suppressor isoform of BIN1 (due to exon 12A skipping event) [80] Another SRSF protein, SRSF2 (also called SC35), is overexpressed in ovarian and neck and head cancers [82,83,84], and plays critical role in cell proliferation and genomic stability with potential link to cancer progression [83]. In head and neck cancer, an exon 11 skipping event in the E-cadherin pre-mRNA results in down-regulation of E-cadherin (a tumor suppressor gene) expression, leading to overexpression of SRSF2 [84]. Previous studies have also proposed *SRSF3* (also known as *SRp20*) and *SRSF6* as proto-oncogenes [85,86]. Overexpression of SRSF3 is observed in human cervical, breast, ovarian, stomach, skin, thyroid, kidney and bladder cancers [85,86,87], while up-regulation of SRSF6 has been implicated in lung [88] and colon [88] cancers. Up-regulation of SRSF2, 3, 5 and 6 has been associated with enhanced expression of anti-apoptotic protein isoforms, including caspase-8L, caspase-9b, c-FLIPs, Bcl-xL, and MNK-2b [81]. Knockdown of *SRSF3* induces cell apoptosis in various types of cancer cells [85,89]. Additionally, suppression of SRSF3 leads to increased expression of the P53β isoform (encoded by an alternatively spliced variant that includes exon i9, an aberrant exon residing in intron 9) and enhanced cellular senescence [90]. These findings provide evidence that SRSF3 is an oncoprotein promoting proliferation/survival and anti-apoptosis in cancer cells.

HNRNP proteins have likewise been implicated as splicing factor oncoproteins [91] where HNRNP A1 and A2 are overexpressed in many cancers [92,93,94]. Small interfering RNA (siRNA) experiments have demonstrated that suppression of HNRNP A1/A2 causes enhanced apoptosis of cancer cells, suggesting that HNRNP A1 and A2 function as anti-apoptotic proteins promoting cell proliferation/survival [93]. HNRNP A2 (as well as B1 and K) has been associated with enhanced expression of anti-apoptotic variants of *BIN1* and *capase-9*, and decreased expression of pro-apoptotic variant *Bcl-xS* [81]. HNRNP A1 and A2 overexpression is linked to a metabolic shift from oxidative phosphorylation to aerobic glycolysis (Warburg effect). These two splicing factors facilitate the AS of *PKM* pre-mRNA by favoring the generation of the *PKM2* variant (promotes aerobic glycolysis) over the *PKM1* variant (promotes oxidative phosphorylation) [95]. Lastly, HNRNP F is overexpressed in primary and metastatic colorectal cancers, indicating a potential role by this splicing factor in early-stage tumor progression and as a potential cancer biomarker [96].

### 3.2. Mutations in Splicing Factors

Mutations in cis-acting splicing factor binding sequences and mutations in critical components of the spliceosome may also lead to cancer development and progression. Genomic analysis by pairing DNA and RNA-Seq data from The Cancer Genome Atlas (TCGA) has revealed that somatic mutations in the splice donor/acceptor sites, last-base exon (LBE) sites, and ESEs and ESSs can affect RNA splicing in tumor cells [97,98,99]. Somatic mutations in LBE sites were enriched in tumor suppressor genes while the mutations in ESE and ESS sites were often involved in oncogenes [100]. These results suggest that mutations in cis-acting splicing factor binding sites can cause aberrant AS of specific oncogenes/tumor suppressor genes and may functionally contribute to cancer development/progression. Next-generation sequencing has uncovered numerous somatic mutations involving splice factors in myeloid neoplasms [101]. Spliceosome mutations are found to reside in more than half of the patients with myelodysplastic syndromes (MDS), and these mutations are likely contributing to disease pathogenesis [102]. Mutations in splicing factors SF3B, SRSF2 and U2AF1 are frequently detected among MDS patients. Previous studies have shown that mutations in SF3B, SRSF2 and U2AF1 occur in 20%, 12% and 6% of MDS patients, respectively [103,104,105]. In addition, mutations in genes involving the splicing machinery have been associated with decreased survival and pathogenesis in acute myeloid leukemia (AML) [106]. Mutations in genes encoding splicing factors (mostly in SRSF2, SF3B1, U2AF1 and ZRSR2) and/or proteins involved in chromatin remodeling (ASXL1, STAG2, BCOR, MLLPTD, EZH2 and PHF6) occur in 18% of AML patients. Poor clinical outcomes are observed in AML patients harboring these mutations, especially patients carrying concurrent mutations in both spliceosome-chromatin and TP53 [106]. DEAD-box polypeptide 41 (DDX41) belongs to RNA helicase family, and plays an important role in spliceosome assembly [107]. DDX41 mutations have been identified in ~3% of inherited hematologic malignancies [108], and the defective DDX41 have been implicated in promoting exon skipping or exon retention in dozens of genes [109]. Mutations in SRSF2 result in mutant proteins that promote or suppress the inclusion of exons containing C- or G-rich motifs [110] and alter the splicing patterns of hundreds of genes [111]. In addition, SRSF2 mutations cause the alternatively splicing of *EZH2* and *BCOR* (two commonly mutated genes in MDS) [112] and have been suggested as a driver of MDS pathogenesis.

## 4. Aberrant mRNA Splicing and Cancer Drug Resistance

### 4.1. BCR-ABL Splice Variant and Imatinib Resistance

**Figure 3:**
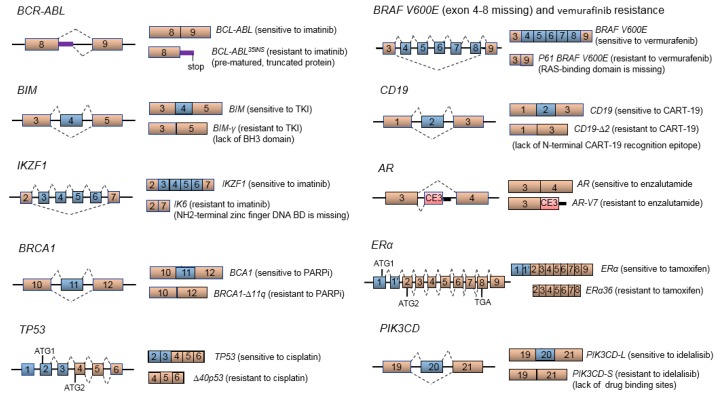
Aberrant mRNA splice variants in cancer drug resistance. AS events involving oncogenes, *BCR-ABL*, *BIM*, *IKZF1*, *BRCA1*, *TP53*, *BRAF*, *CD19*, *AR*, *ER* and *PIK3CD*, leading to resistance to targeted therapies, chemotherapy, hormone therapy or immunotherapy [121,122,123,124,125,126,127]. ATG1: Start codon for full-length TP53 and ERα. ATG2: Start codon for *∆40p53* and *ERα36* splice variants. TGA: Stop codon for *ERα36* splice variant. See text for additional references.

Imatinib is a tyrosine kinase inhibitor (TKI) that targets the oncogenic BCR-ABL fusion protein in chronic myelogenous leukemia (CML). CML patients who receive imatinib therapy and exhibit an earlier/deeper molecular response have more favorable clinical outcomes, including prolonged relapses-free and overall survival rates [113,114]. However, ~20% of CML patients that initially respond to imatinib will develop resistance primarily due to point mutations within the kinase domain of the *BCR-ABL* gene. In addition to these mutations, alternatively spliced *BCR-ABL* variants are known to exist, and the *BCR-ABL35INS* variant has been associated with poor response to TKI treatment [115,116,117]. *BCR-ABL35INS* retains 35 intronic nucleotides between exons 8 and 9, leading to a frameshift and pre-mature termination of the encoded protein (Figure 3). As a result, the remaining portion of the kinase domain on the truncated BCR-ABL35INS protein is kinase-inactive [118,119] and 3-dimensional modeling suggests a global conformational shift of the protein that is associated with poor TKI binding [120]. The mechanism of resistance and clinical significance of this splice variant remains unclear and controversial.

### 4.2. BCL2-Like 11 (BIM or BCL2L11) Splice Variant and TKI Resistance

One of the proposed mechanisms of imatinib-induced apoptosis in leukemic cells involves increased transcription of the *BCL2-like 11* (*BIM* or *BCL2L11*) gene and post-translational activation of BIM protein [128]. Failure of CML patients to respond to imatinib therapy has been linked to point mutations in the target protein BCR-ABL and/or AS of the BCR-ABL pre-mRNA (see Section 4.1). Interestingly, another potential mechanism responsible for imatinib resistance may involve AS of *BIM*. Multiple *BIM* splice variants have been identified including *BIM-γ*, arising from an exon 4-to-exon 3 switch due to an intronic deletion polymorphism [124]. Genomic analysis has revealed that expression of the *BIM-γ* splice variant is correlated with imatinib-resistant CML, as well as resistance to other TKIs [124]. Exon 4 of *BIM* encodes a BH3 domain, which is essential for the pro-apoptotic function of BIM. The exon 4-to-exon 3 switch introduces a polyadenylation signal sequence leading to premature translation termination and loss of the BH3 domain [129,130]. Indeed, BCR-ABL-positive CML and EGFR mutation-positive non-small cell lung cancer (NSCLC) cells expressing BIM-γ exhibit resistance to imatinib and gefitinib (EGFR TKI), respectively [124]. However, this drug resistance can be bypassed by treating CML and NSCLC with BH3 mimetic drugs [124]. More recently, a systematic antisense splice-switching oligonucleotide (ASO) ‘walking’ screen identified 67 ASOs that corrected aberrant *BIM* splicing by preventing the exon 4-to-exon 3 switch. This led to a restoration of TKI sensitivity and re-sensitized leukemic cells to imatinib-induced apoptosis [130].

### 4.3. BRCA Splice Variants Leading to PARP Inhibitor or Cytotoxic Drug Resistance

*BRCA1* and *BRCA2* encode tumor suppressor proteins that are required for homologous recombination (HR)-mediated repair of double-strand DNA (dsDNA) breaks [131,132]. Germline loss-of-function mutations in *BRCA1* or *BRCA2* have been implicated in increased risk of breast and ovarian cancers [133,134]. In the absence of either BRCA1 or BRCA2 activity, poly (ADP-ribose) polymerase (PARP), which functions as an enzyme to repair single-strand DNA (ssDNA) breaks through base excision repair, is thought to be an essential safeguard for maintaining genome integrity. Consequently, PARP inhibitors (PARPi) may serve as an effective agent to induce ‘synthetic lethality’ of tumor cells with dysfunctional BRCA1 and/or BRCA2 [135,136,137]. Although PARPi therapy has been demonstrated to efficiently sensitize BRCA1/2 mutation-associated cancers and improve survival in patients, not all patients respond to therapy and some patients developed drug resistance after initial favorable response [138,139].

Aberrant pre-mRNA splicing has been implicated as a potential mechanism responsible for the development of PARPi resistance. Inactivating mutations in exon 11 of *BRCA1* account for ~30% of breast and ovarian cancers [140,141,142]. A recent study has revealed that a *BRCA1-∆11q* splice variant, where the majority of exon 11 is skipped and consequently bypasses any inactivating mutations in this region, promotes partial resistance to PARPi therapy (Figure 3) [125]. Interestingly, PARPi resistance can be reversed with a small molecule inhibitor P1-B that suppresses the U2 snRNP spliceosome machinery, leading to a silencing of the splicing event responsible for generating *BRCA1-∆11q* and re-sensitization of cancer cells to PARPi treatment [125]. In addition, aberrant splicing of *BRCA2* and chemoresistance has been reported in a recent study [143]. A novel splice variant *BRCA2^∆E5+7^*, missing exons 5 and 7, encodes an in-frame protein isoform with an internal deletion of 55 amino acids compared to wild-type BCRA2. Expression of this aberrant isoform has been associated with the acquisition of resistance to the DNA cross-linking drug mitomycin C [143].

### 4.4. TP53 Splice Variants and Cisplatin Resistance

Alternative splicing of the *TP53* pre-mRNA produces at least 12 splice variants [144]. Expression of specific P53 isoforms, such as P53β, ∆40p53, and ∆133p53 [145,146,147], has been associated with tumor progression, clinical response and prognosis [144]. P53β, ∆40p53, and ∆133p53 are small molecular weight isoforms compared to full-length wild-type P53. In response to the DNA alkylating agent cisplatin, P53β and ∆40p53 have been demonstrated to differentially regulate downstream signaling [146]. Namely, the P53β isoform stimulates transcription of the *P21* and *PUMA* genes in a wild-type P53-dependent manner, while the ∆40p53 isoform has the opposite effect in melanoma cells. The latter appears to contribute to resistance to cisplatin therapy [146].

### 4.5. BRAF V600E Splice Variant and Vermurafenib Resistance

The *BRAF* gene encodes a serine/threonine kinase that is a critical component in the RAS-RAF-MEK-ERK-MAPK pathway in response to growth signaling [148]. Previous studies have revealed that more than 60% of malignant melanoma patients carry mutations in the *BRAF* gene, and all mutations are localized within the kinase domain [148]. The BRAF V600E mutation, resulting in a single valine-to-glutamate substitution, accounts for the majority of the BRAF mutations among melanoma patients [148]. Vemurafenib is a BRAF inhibitor specifically targeting melanomas carrying the BRAF V600E mutation. BRAF inhibitors (including vemurafenib) bind to the ATP-binding site of the 90 kDa BRAF (V600E) mutant to inhibit kinase activity [149,150,151]. Therefore, BRAF (V600E) serves as a molecular target and BRAF inhibitors have remarkable clinical value in treating melanomas [152].

**Figure 4:**
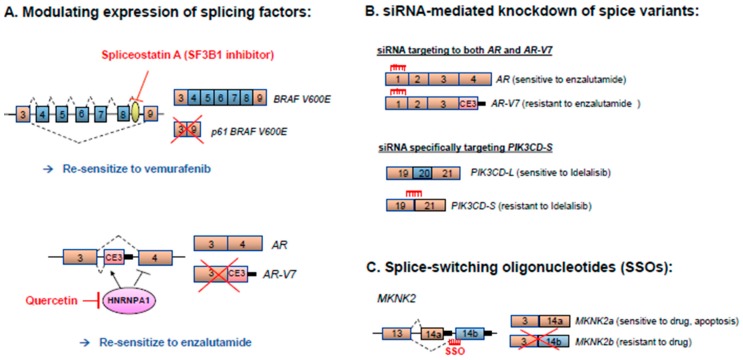
Different re-sensitization approaches to reverse RNA splicing events causing drug resistance. (**A**) Top panel: SiRNA-mediated knockdown of oncogenic splice variants. SiRNAs targeting exon 1of *AR-V7* variant. Bottom panel: SiRNA targeting the junction of exons 19 and 21 to specifically inhibit expression of *PIK3CD-S*. (**B**) Splice switching oligonucleotides (SSO) as a strategy to interfere with aberrant splicing. Example illustrated is an SSO (or splice switching anti-sense oligonucleotide, ASO) bound to the intron-exon junction to prevent splice generation of the *MKNK2-2b* variant, thereby re-sensitizing cancer cells to drug treatment. (**C**) Pharmacologic inhibition of splicing factors. Spliceostatin A (SF3B1 inhibitor) and quercetin (HNRNPA1 inhibitor) suppress formation of *p61 BRAF V600E* and *AR-V7* splice variants, respectively. These approaches will induce re-sensitization of cancer cells to drug treatments. See text for corresponding references.

However, a 61kDa BRAF (V600E) isoform, encoded by a splice variant with an exon 4–8 skipping event, is expressed in melanoma patients exhibiting resistance to vemurafinib (Figure 3) [126,153]. Exons 4 through 8 of *BRAF* encode the RAS-binding domain (RBD), and the multi exon skipping event results in a p61 BRAF V600E isoform missing the RBD. This leads to constitutive isoform dimerization in a RAS-independent manner, conferring resistance to vemurafenib [126]. Interference of the pre-mRNA splicing machinery has been proposed as a therapeutic strategy to overcome vemurafenib resistance. Spliceostatin A and its analog meayamycin B target splicing factor SF3B1 and inhibit formation of the *p61 BRAF V600E* splice variant, which in turn re-sensitizes therapy-resistant melanomas to vemurafenib as demonstrated in both in vitro and in vivo models (Figure 4A) [154].

### 4.6. CD19 Splice Variant and CART-19 Immunotherapy Resistance in B-Cell Acute Lymphoblastic Leukemia

Immunotherapy has been demonstrated as an effective approach for treating relapsed B-cell acute lymphoblastic leukemias (B-ALLs). For example, patients with B-cell malignancies are being treated with adoptive T cells expressing a chimeric antigen receptor (CAR) targeting the CD19 epitope [155,156]. CD19 is a cell surface protein that triggers activation of PI3K and LYN signaling in neoplastic B-cells [157], and has been implicated in B-cell neoplasms [158,159,160]. CAR T-cell immunotherapy has been documented to successfully treat B-ALL with an overall 70% to 90% remission rate [161]. However, relapse has been observed in CAR T-cell treated patients exhibiting an apparent loss of CD19 surface expression on malignant cells. One of the proposed mechanisms underlying loss of CD19 is AS. Skipping of exon 2 during processing of the CD19 pre-mRNA (leading to the formation of the *CD19-∆2* variant) results in an N-terminally truncated CD19 protein lacking the CAR recognition site, which prevents CAR T-cell targeting/killing of B-ALL cells [127]. Previous studies have shown that the loss of the CD19 epitope occurs in 10% to 20% of the pediatric B-ALL patients [162,163], contributing to immunotherapy resistance among these patients. One possible strategy for overcoming this immunotherapy resistance is to develop a novel CART-19 that targets alternative CD19 ectodomains.

### 4.7. Truncated AR Variants and Androgen-Independent/Refractory Prostate Cancer Disease

The androgen receptor (AR) is a critical therapeutic target for treating advanced prostate cancer. Aberrant *AR* splicing patterns in prostate cancer has been identified as a key mechanism leading to acquired resistance to androgen ablation therapy. Several *AR* splice variants have been implicated in castration-resistance (e.g., androgen-refractory/independent) in in vitro cell models, and in vivo xenograft and transgenic mouse models [47]. Skipping of exons 2, 3 or 7, or retention of intron 6 during *AR* pre-mRNA processing are well-recognized aberrant splicing events [47]. Truncated AR protein isoforms, resulting from the exon skipping events, lack the ligand-binding domain (LBD) and translocate to the nucleus in an androgen-independent manner [164,165] resulting in constitutive expression of AR-target genes [166].

*AR-V7*, one of the best characterized AR splice variants, is missing exons 4–7 and prominently expressed in hormone-refractory prostate cancer (Figure 2) [165,167]. The *AR-V7* variant encodes a functional protein lacking the LBD, a crucial target for androgen ablation therapy. Consequently, patients with *AR-V7*-expressing prostate cancer (Figure 3) exhibit resistance to the antiandrogen drugs enzalutamide (AR antagonist) and abiraterone (inhibitor of steroidogenic enzyme CYP17A1) [121]. In addition, overexpression of the *AR-V7* isoform has been correlated with poorer patient survival and higher recurrence rates [121,165]. In the prostate cancer cell line 22Rv1 that expresses the full-length *AR* mRNA and a truncated *AR* splice variant, siRNA-mediated knockdown of both full-length and truncated variant, but not knockdown of full-length alone, effectively suppresses androgen-independent cell proliferation and initiates cell apoptosis [168]. It has also been shown that suppression of HNRNPA1 by quercetin can prevent the generation of *AR-V7* and re-sensitize the prostate cancer cells to enzalutamide (Figure 4A) [169,170]. A proteomic approach has identified HNRNPA1 and EF-1α as molecular targets of quercetin [171]. Quercetin binding to EF-1α disrupts formation of an EF-1α-GTP-tRNA complex and/or prevents this complex from interacting with ribosomes [172]. The cooperative effects of quercetin to inhibit HNRNPA1 nuclear translocation and disrupt the tRNA machinery may explain how this small molecule inhibitor suppresses the generation of aberrant splice variant *AR-V7*.

### 4.8. ER Splice Variants and Tamoxifen Resistance

Estrogen is essential for growth and development of the mammary glands and plays an important role in the development and progression of breast cancer. The estrogen receptor (ER) is comprised of two isoforms ERα and ERβ, which are encoded by the *ESR1* and *ESR2* genes, respectively [173]. ERα splice isoforms of full-length ERα (ERα66) have been identified, ERα46 and ERα36 [174,175]. ERα66 (consisting of 595 amino acids) contains the constitutive activation function (AF-1) and hormone-dependent activation function (AF-2) domains, as well as a DNA binding domain (DBD) [175]. ERα46 is missing the first 173 amino acids of ERα66 due to alternative promoter usage, which results in a truncated protein isoform lacking the AF-1 domain [174]. Splice isoform ERα36 lacks the AF-1 and part of the AF-2 domains, and has a unique 27-amino-acid C-terminus that replaces the last 138 amino acids encoded by exons 7 and 8 of ERα66 [174]. Genomic and nongenomic estrogen signaling are mediated by ERα66 and ERα36, respectively [174,175]. ERα36 is highly expressed in ERα-negative breast cancer, and overexpression of ERα36 causes a decrease in ERα66 [176]. Conversely, it has also been reported that expression of ERα66 negatively regulates the expression of ERα36 transcript [177]. Tamoxifen has been successfully used in the treatment of ER-positive breast cancer patients for decades. It has been reported that patients with breast tumors expressing ERα36 benefit less from tamoxifen therapy compared to those with breast tumors expressing ERα66 [178,179]. Interestingly, ERα36 levels were found co-localized and positively correlated with the expression of stem cell marker aldehyde dehydrogenase 1A1 (ALDH1A1) in tamoxifen-resistant breast cancers [180]. This AS-driven tamoxifen resistance may be overcome by suppressing ERα36 using shRNA [181], ALDH1 inhibitors, or ERα36-specific antibody [180].

### 4.9. PIK3CD Splice Variant and Idelalisib Resistance

PI3K plays a central role in PI3K/AKT/mTOR signaling, and activating mutations in this pathway are associated with cancer proliferation, survival, invasion and migration/metastasis [182]. An integrative genomic analysis has revealed >2000 differential RNA splicing events between African American (AA) and European American (EA) prostate cancers [122]. Specific splice variants of *PIK3CD*, *TSC2*, and *RASGRP2* are associated with a more aggressive oncogenic phenotype in AA prostate cancer cells, which has been hypothesized as an underlying mechanism for prostate cancer health disparities between AA and EA men [122]. Notably, both *PIK3CD-L* (long splice variant containing all 24 exons; see Figure 3) and *PIK3CD-S* (short variant with all exons except exon 20) are expressed in AA prostate cancer, while *PIK3CD-L* is the major variant expressed in EA prostate cancer. In vitro and in vivo assays have demonstrated that the *PIK3CD-S* splice variant is associated with more aggressive oncogenic properties (enhanced proliferative, invasive and metastatic capabilities) [122]. An siRNA designed to specifically target the junction of exons 19 and 21 effectively inhibits the proliferative- and invasive-rendering phenotype of the *PIK3CD-S* variant (Figure 4B) [122]. Skipping of exon 20 in *PIK3CD-S* results in a PI3Kδ short isoform lacking 56 amino acids that are part of the hinge region of the catalytic kinase domain. The 56 amino acid region also contains critical amino acids for the docking of idelalisib, a specific inhibitor of PI3Kδ [183]. In vitro and in vivo experiments reveal that the PIK3CD-S variant encodes a PI3Kδ isoform resistant to inhibition by idelalisib and wartmannin (non-selective pan-PI3K inhibitor) [122]. Notably, idelalisib is currently used in the treatment of B-cell malignancies such as chronic lymphocytic leukemia (CLL) [184]. However, 20% to 50% of CLL patients have poor response to idelalisib [185,186,187]. Our analysis of publicly available TCGA RNA-Seq data suggests that a high *PIK3CD-S/PIK3CD-L* mRNA expression ratio is associated with poor survival of patients with prostate, breast and colon cancers [122]. It will also be of interest to ascertain in future studies whether high expression of the *PIK3CD-S* variant is coupled to idelalisib resistance.

## 5. Therapeutic Strategies for Correcting Aberrant Splicing Errors

**Figure 5:**
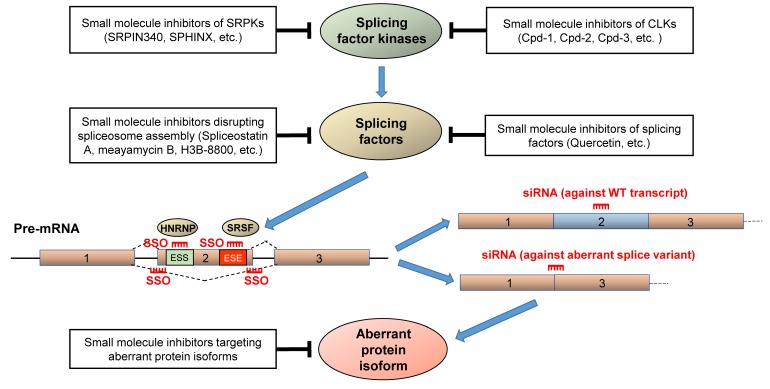
Potential therapeutic approaches to correct splicing errors. Small molecule inhibitors (Cpd-1, Cpd-2, Cpd-3, SRPIN340, SPHINX) can be used to block the activity of splicing factor kinases (CLKs and SRPKs) thereby reversing aberrant mRNA splicing. Small molecule inhibitors of splicing factors (Spliceostatin A, Meayamycin B, H3B-8800, Quercetin) can reverse aberrant splicing by blocking spliceosome assembly or directly targeting splicing factors. Splice switching oligonucleotides (SSOs) can prevent SRSF and HNRNP proteins from interacting with exon splicing enhancers (ESEs) and exonic splicing silencers (ESSs) located on the pre-mRNA, respectively. SSOs targeting exon-intron junctions can disrupt alternative splicing. Future small molecules can be developed to specifically target aberrant protein isoforms. All of these strategies may be used to correct splicing errors and overcome drug resistance. See text for corresponding references.

Aberrant mRNA splicing often encodes protein isoforms with distinct properties, and in some cases (as described above), promotes drug/therapy resistance. Oncogenic splicing errors may be mitigated by directly targeting the aberrant protein isoforms, or targeting their upstream splicing regulators using pharmacologic or molecular approaches. Figure 5 summarizes the various approaches to correct aberrant splicing errors and to re-sensitize cancer cells to therapeutics. Small molecule inhibitors have been successfully employed to target splicing factors, splicing factor kinases, or upstream splicing regulators and to alter mRNA splicing patterns. Likewise, siRNAs and SSOs have also been applied to specifically target and disrupt the splicing regulatory sequences or splicing factor/RNA interactions at the pre-mRNA level [188,189]. SF3B1-targeting agents, such as spliceostatins, pladienolides and herboxidienes, are small molecule inhibitors used to disrupt the early stage of spliceosome assembly [188]. For example, spliceostatin A, meayamycin B and sudemycins (spliceostatin A analogs), and E7107 (pladienolide analog) bind SF3B1 to prevent formation of an U2 snRNP-SF3B1 complex with pre-mRNAs [188,190]. Spliceostatin A and meayamycin B have been shown to correct splicing errors by inhibiting exon skipping of *BRAF V600E* and overcome p61 BRAF V600E-driven vemurafenib resistance (Figure 4A) [154]. H3B-8800, another splicing modulator targeting SF3B1, inhibits expression of aberrant splice variant *MAP3K7* in a dose-dependent manner and preferentially kills epithelial and hematologic malignancies expressing mutant spliceosome components [191]. Besides splicing factors (Figure 4A), the targeting of splicing factor kinases, such as the CDC2-like kinases (CLKs) and serine-arginine protein kinases (SRPKs), has emerged as a potential therapy to reverse aberrant RNA splicing [188,189]. For example, small molecule inhibitor SRPIN340 blocks SRPK1-mediated phosphorylation of SRSF1 [192], leading to splice switching of pro-angiogenic *VEGFA165* to anti-angiogenic *VEGFA165b* in prostate cancer and leukemic cells [193,194]. SPHINX, a new generation SPRK1 inhibitor, likewise promotes splice switching of VEGFA165 to VEGFA165b to inhibit tumor growth in vivo [195]. The small molecule inhibitors of the CLKs (Cpd-1, Cpd-2 and Cpd-3) significantly suppress phosphorylation of splicing factors SRSF1, SRSF4 and SRSF6, thereby altering the splicing pattern of S6K pre-mRNAs, reducing cell proliferation and promoting cell apoptosis [196]. In addition, oligonucleotide-based therapies have been demonstrated as effective strategies for targeting wild-type or aberrant splice variants with high selectivity/specificity. SiRNAs have been used to specifically target AR, *AR-V7*, PIK3CD-L and PIK3CD-S splice variants [122,164,168], while SSOs have been leveraged to modify splicing of *MDM4*, STAT3, KRAS, and *BCL2L1* [197,198,199,200]. Both siRNA- and SSO-mediated strategies have successfully suppressed in vitro and/or in vivo tumor cell growth.

## 6. Concluding Remark and Perspectives

Accumulating evidence suggests that aberrant splicing mechanisms likely play an essential role in cancer development and progression. In this review, we have highlighted the critical consequences of splicing factor dysfunction and aberrant mRNA splicing in promoting/driving tumorigenesis and drug resistance. A number of cancer-specific splice variants have been discovered, including but not limited to *BCL2L1*, *FAS*, *HRAS*, *Cyclin D1*, *CASP2*, *CD44*, *TMPRSS-EGR*, *FGFR2*, *VEGF*, *AR*, and *KLF6-SV1*. Moreover, aberrant splicing may be an intrinsic mechanism leading to therapy resistance. Aberrant splicing through intron retention (*BCR-ABL35INS*) or exon-skipping events (*BIM-γ*, *IK6*, *BRCA1-∆11q*, *p61BRAF V600E*, *CD19-∆4*, *AR-V7* and *PIK3CD-S*) results, in some instances, to insensitivity to targeted therapies due to structural changes in drug-targeting domains. There are numerous additional examples of specific splice variants associated with greater cancer aggressiveness, but links between these variants and drug resistance have yet to be fully explored. For example, fusion of the coding region of *ERG* with the promoter region of the *TMPRSS* gene is observed in approximately half of all prostate cancers. This fusion event leads to ERG protein overexpression and chemotherapy resistance to the taxanes [201]. Given that the *TMPRSS2–ERG+72bp* splice variant (which includes 72 bp exon 11) is associated with more aggressive prostate cancer compared to variants excluding the 72 bp exon 11 (Figure 2) [48,49,202], it will be of interest to determine if the former variant is also associated with greater chemoresistance compared to the latter variants. It is clear that the identification of aberrant splicing patterns and understanding their mechanistic consequences will be crucial for developing novel therapeutic strategies to overcome splicing-driven drug resistance and/or re-sensitize cancer cells to drug therapy. Potential therapeutic re-sensitization approaches include oligonucleotide-mediated gene therapy (siRNAs or SSOs) [203], modulating function or expression of splicing factors using small molecule inhibitors or siRNAs (see Figure 5 for the summary of potential re-sensitization approaches).

The establishment of in vivo animal models will likewise be crucial for the development of new strategies to overcome therapy resistance driven by oncogenic splice variants. A major hurdle is the low conservation (20% to 25%) of alternative splicing events observed in humans and mice [204]. Consequently, in vivo splice variant-specific knockout mouse models should only be considered when RNA splicing for a particular gene is conserved between the two species. An alternative strategy may be to employ transgenic mouse models [205] to facilitate our understanding of in vivo aberrant splicing in cancer development and progression.

Finally, systematic and genome-wide mapping of splicing events in cancers will further our understanding of mechanisms promoting drug resistance. Recent genomics studies using next-generation sequencing (NGS) approaches have revealed global alternative splicing profiles in multiple types of cancers [206,207,208,209,210]. Notably, these studies have highlighted the prognostic value of aberrant splice variants in NSCLC [206], ovarian cancer [207], breast cancer [208], uveal melanoma [209] and glioblastoma [210]. Most recently, a comprehensive analysis of alternative splicing mapping across 32 TCGA cancer types has revealed tumor-specific splicing patterns [211]. Approximately 251,000 neojunctions (novel exon-exon junctions) with an average of ~930 neojunctions per cancer sample have been identified. The identification of a new class of neoantigens could be leveraged for designing novel immunotherapeutic interventions (i.e., CAR T-cell therapy) to overcome splicing-driven immunotherapy resistance. Advancements in cancer genomics will pave a new avenue for developing novel diagnostic and therapeutic strategies towards cancer precision medicine.

## References

[1] Adams M.D., Kerlavage A.R., Fleischmann R.D., Fuldner R.A., Bult C.J., Lee N.H., Kirkness E.F., Weinstock K.G., Gocayne J.D., White O. (1995). Initial assessment of human gene diversity and expression patterns based upon 83 million nucleotides of cDNA sequence. Nature.

[2] Hallegger M., Llorian M., Smith C.W. (2010). Alternative splicing: Global insights. FEBS J..

[3] Pan Q., Shai O., Lee L.J., Frey B.J., Blencowe B.J. (2008). Deep surveying of alternative splicing complexity in the human transcriptome by high-throughput sequencing. Nat. Genet..

[4] Wang E.T., Sandberg R., Luo S., Khrebtukova I., Zhang L., Mayr C., Kingsmore S.F., Schroth G.P., Burge C.B. (2008). Alternative isoform regulation in human tissue transcriptomes. Nature.

[5] Blencowe B.J. (2006). Alternative splicing: New insights from global analyses. Cell.

[6] Sakharkar M.K., Chow V.T., Kangueane P. (2004). Distributions of exons and introns in the human genome. In Silico Biol..

[7] Stamm S., Ben-Ari S., Rafalska I., Tang Y., Zhang Z., Toiber D., Thanaraj T.A., Soreq H. (2005). Function of alternative splicing. Gene.

[8] Castle J.C., Zhang C., Shah J.K., Kulkarni A.V., Kalsotra A., Cooper T.A., Johnson J.M. (2008). Expression of 24,426 human alternative splicing events and predicted *cis* regulation in 48 tissues and cell lines. Nat. Genet..

[9] Clark T.A., Schweitzer A.C., Chen T.X., Staples M.K., Lu G., Wang H., Williams A., Blume J.E. (2007). Discovery of tissue-specific exons using comprehensive human exon microarrays. Genome Biol..

[10] De la Grange P., Gratadou L., Delord M., Dutertre M., Auboeuf D. (2010). Splicing factor and exon profiling across human tissues. Nucleic Acids Res..

[11] Kwan T., Benovoy D., Dias C., Gurd S., Provencher C., Beaulieu P., Hudson T.J., Sladek R., Majewski J. (2008). Genome-wide analysis of transcript isoform variation in humans. Nat. Genet..

[12] Tang F., Barbacioru C., Wang Y., Nordman E., Lee C., Xu N., Wang X., Bodeau J., Tuch B.B., Siddiqui A. (2009). mRNA-Seq whole-transcriptome analysis of a single cell. Nat. Meth..

[13] Barash Y., Calarco J.A., Gao W., Pan Q., Wang X., Shai O., Blencowe B.J., Frey B.J. (2010). Deciphering the splicing code. Nature.

[14] Black D.L. (2003). Mechanisms of alternative pre-messenger RNA splicing. Annu. Rev. Biochem..

[15] Bland C.S., Wang E.T., Vu A., David M.P., Castle J.C., Johnson J.M., Burge C.B., Cooper T.A. (2010). Global regulation of alternative splicing during myogenic differentiation. Nucleic Acids Res..

[16] Johnson M.B., Kawasawa Y.I., Mason C.E., Krsnik Z., Coppola G., Bogdanovic D., Geschwind D.H., Mane S.M., State M.W., Sestan N. (2009). Functional and evolutionary insights into human brain development through global transcriptome analysis. Neuron.

[17] Trapnell C., Williams B.A., Pertea G., Mortazavi A., Kwan G., van Baren M.J., Salzberg S.L., Wold B.J., Pachter L. (2010). Transcript assembly and quantification by RNA-Seq reveals unannotated transcripts and isoform switching during cell differentiation. Nat. Biotechnol..

[18] Kornblihtt A.R. (2005). Promoter usage and alternative splicing. Curr. Opin. Cell Biol..

[19] Cartegni L., Chew S.L., Krainer A.R. (2002). Listening to silence and understanding nonsense: Exonic mutations that affect splicing. Nat. Rev. Genet..

[20] Rajan P., Elliott D.J., Robson C.N., Leung H.Y. (2009). Alternative splicing and biological heterogeneity in prostate cancer. Nat. Rev. Urol..

[21] David C.J., Manley J.L. (2010). Alternative pre-mRNA splicing regulation in cancer: Pathways and programs unhinged. Genes Dev..

[22] Venables J.P., Klinck R., Koh C., Gervais-Bird J., Bramard A., Inkel L., Durand M., Couture S., Froehlich U., Lapointe E. (2009). Cancer-associated regulation of alternative splicing. Nat. Struct. Mol. Biol..

[23] Venables J.P. (2006). Unbalanced alternative splicing and its significance in cancer. Bioessays.

[24] Skotheim R.I., Nees M. (2007). Alternative splicing in cancer: Noise, functional, or systematic?. Int. J. Biochem. Cell Biol..

[25] Omenn G.S., Yocum A.K., Menon R. (2010). Alternative splice variants, a new class of protein cancer biomarker candidates: Findings in pancreatic cancer and breast cancer with systems biology implications. Dis. Mark..

[26] Srebrow A., Kornblihtt A.R. (2006). The connection between splicing and cancer. J. Cell Sci..

[27] Germann S., Gratadou L., Dutertre M., Auboeuf D. (2012). Splicing programs and cancer. J. Nucleic Acids.

[28] Dutertre M., Vagner S., Auboeuf D. (2010). Alternative splicing and breast cancer. RNA Biol..

[29] Venables J.P. (2004). Aberrant and alternative splicing in cancer. Cancer Res..

[30] Ghigna C., Valacca C., Biamonti G. (2008). Alternative splicing and tumor progression. Curr. Genom..

[31] Boise L.H., Gonzalez-Garcia M., Postema C.E., Ding L., Lindsten T., Turka L.A., Mao X., Nunez G., Thompson C.B. (1993). Bcl-x, a bcl-2-related gene that functions as a dominant regulator of apoptotic cell death. Cell.

[32] Akgul C., Moulding D.A., Edwards S.W. (2004). Alternative splicing of Bcl-2-related genes: Functional consequences and potential therapeutic applications. Cell. Mol. Life Sci..

[33] Minn A.J., Boise L.H., Thompson C.B. (1996). Bcl-x(S) anatagonizes the protective effects of Bcl-x(L). J. Biol. Chem..

[34] Olopade O.I., Adeyanju M.O., Safa A.R., Hagos F., Mick R., Thompson C.B., Recant W.M. (1997). Overexpression of BCL-x protein in primary breast cancer is associated with high tumor grade and nodal metastases. Cancer J. Sci. Am..

[35] Takehara T., Liu X., Fujimoto J., Friedman S.L., Takahashi H. (2001). Expression and role of Bcl-xL in human hepatocellular carcinomas. Hepatology.

[36] Mercatante D.R., Mohler J.L., Kole R. (2002). Cellular response to an antisense-mediated shift of Bcl-x pre-mRNA splicing and antineoplastic agents. J. Biol. Chem..

[37] Bouillet P., O’Reilly L.A. (2009). CD95, BIM and T cell homeostasis. Nat. Rev. Immunol..

[38] Cheng J., Zhou T., Liu C., Shapiro J.P., Brauer M.J., Kiefer M.C., Barr P.J., Mountz J.D. (1994). Protection from Fas-mediated apoptosis by a soluble form of the Fas molecule. Science.

[39] Cascino I., Fiucci G., Papoff G., Ruberti G. (1995). Three functional soluble forms of the human apoptosis-inducing Fas molecule are produced by alternative splicing. J. Immunol..

[40] Poh T.W., Pervaiz S. (2005). LY294002 and LY303511 sensitize tumor cells to drug-induced apoptosis via intracellular hydrogen peroxide production independent of the phosphoinositide 3-kinase-Akt pathway. Cancer Res..

[41] Tamm C., Zhivotovsky B., Ceccatelli S. (2008). Caspase-2 activation in neural stem cells undergoing oxidative stress-induced apoptosis. Apoptosis.

[42] Bonzon C., Bouchier-Hayes L., Pagliari L.J., Green D.R., Newmeyer D.D. (2006). Caspase-2-induced apoptosis requires bid cleavage: A physiological role for bid in heat shock-induced death. Mol. Biol. Cell.

[43] Solier S., Logette E., Desoche L., Solary E., Corcos L. (2005). Nonsense-mediated mRNA decay among human caspases: The caspase-2S putative protein is encoded by an extremely short-lived mRNA. Cell Death Differ..

[44] Droin N., Beauchemin M., Solary E., Bertrand R. (2000). Identification of a caspase-2 isoform that behaves as an endogenous inhibitor of the caspase cascade. Cancer Res..

[45] Martinet W., Knaapen M.W., De Meyer G.R., Herman A.G., Kockx M.M. (2003). Overexpression of the anti-apoptotic caspase-2 short isoform in macrophage-derived foam cells of human atherosclerotic plaques. Am. J. Pathol..

[46] Parent N., Sane A.T., Droin N., Bertrand R. (2005). Procaspase-2S inhibits procaspase-3 processing and activation, preventing ROCK-1-mediated apoptotic blebbing and body formation in human B lymphoma Namalwa cells. Apoptosis.

[47] Dehm S.M., Tindall D.J. (2011). Alternatively spliced androgen receptor variants. Endocr. Relat. Cancer.

[48] Wang J., Cai Y., Yu W., Ren C., Spencer D.M., Ittmann M. (2008). Pleiotropic biological activities of alternatively spliced TMPRSS2/ERG fusion gene transcripts. Cancer Res..

[49] Tomlins S.A., Bjartell A., Chinnaiyan A.M., Jenster G., Nam R.K., Rubin M.A., Schalken J.A. (2009). ETS gene fusions in prostate cancer: From discovery to daily clinical practice. Eur. Urol..

[50] Cohen J.B., Broz S.D., Levinson A.D. (1989). Expression of the H-ras proto-oncogene is controlled by alternative splicing. Cell.

[51] Knudsen K.E., Diehl J.A., Haiman C.A., Knudsen E.S. (2006). Cyclin D1: Polymorphism, aberrant splicing and cancer risk. Oncogene.

[52] Inoue K., Fry E.A. (2015). Aberrant expression of cyclin D1 in cancer. Sign. Transduct. Insights.

[53] Qie S., Diehl J.A. (2016). Cyclin D1, cancer progression, and opportunities in cancer treatment. J. Mol. Med..

[54] Knudsen E.S., Witkiewicz A.K. (2017). The Strange Case of CDK4/6 Inhibitors: Mechanisms, Resistance, and Combination Strategies. Trends Cancer.

[55] Li Z., Wang C., Jiao X., Katiyar S., Casimiro M.C., Prendergast G.C., Powell M.J., Pestell R.G. (2008). Alternate cyclin D1 mRNA splicing modulates p27KIP1 binding and cell migration. J. Biol. Chem..

[56] Millar E.K., Dean J.L., McNeil C.M., O’Toole S.A., Henshall S.M., Tran T., Lin J., Quong A., Comstock C.E., Witkiewicz A. (2009). Cyclin D1b protein expression in breast cancer is independent of cyclin D1a and associated with poor disease outcome. Oncogene.

[57] Wang Y., Dean J.L., Millar E.K., Tran T.H., McNeil C.M., Burd C.J., Henshall S.M., Utama F.E., Witkiewicz A., Rui H. (2008). Cyclin D1b is aberrantly regulated in response to therapeutic challenge and promotes resistance to estrogen antagonists. Cancer Res..

[58] Gunthert U., Hofmann M., Rudy W., Reber S., Zoller M., Haussmann I., Matzku S., Wenzel A., Ponta H., Herrlich P. (1991). A new variant of glycoprotein CD44 confers metastatic potential to rat carcinoma cells. Cell.

[59] Sahadevan K., Darby S., Leung H.Y., Mathers M.E., Robson C.N., Gnanapragasam V.J. (2007). Selective over-expression of fibroblast growth factor receptors 1 and 4 in clinical prostate cancer. J. Pathol..

[60] Gnanapragasam V.J., Robinson M.C., Marsh C., Robson C.N., Hamdy F.C., Leung H.Y. (2003). FGF8 isoform b expression in human prostate cancer. Br. J. Cancer.

[61] Woolard J., Wang W.Y., Bevan H.S., Qiu Y., Morbidelli L., Pritchard-Jones R.O., Cui T.G., Sugiono M., Waine E., Perrin R. (2004). VEGF165b, an inhibitory vascular endothelial growth factor splice variant: Mechanism of action, in vivo effect on angiogenesis and endogenous protein expression. Cancer Res..

[62] Pajares M.J., Ezponda T., Catena R., Calvo A., Pio R., Montuenga L.M. (2007). Alternative splicing: An emerging topic in molecular and clinical oncology. Lancet Oncol..

[63] Narla G., DiFeo A., Yao S., Banno A., Hod E., Reeves H.L., Qiao R.F., Camacho-Vanegas O., Levine A., Kirschenbaum A. (2005). Targeted inhibition of the KLF6 splice variant, KLF6 SV1, suppresses prostate cancer cell growth and spread. Cancer Res..

[64] Dhanasekaran S.M., Barrette T.R., Ghosh D., Shah R., Varambally S., Kurachi K., Pienta K.J., Rubin M.A., Chinnaiyan A.M. (2001). Delineation of prognostic biomarkers in prostate cancer. Nature.

[65] Narla G., DiFeo A., Fernandez Y., Dhanasekaran S., Huang F., Sangodkar J., Hod E., Leake D., Friedman S.L., Hall S.J. (2008). KLF6-SV1 overexpression accelerates human and mouse prostate cancer progression and metastasis. J. Clin. Investig..

[66] Maglic D., Stovall D.B., Cline J.M., Fry E.A., Mallakin A., Taneja P., Caudell D.L., Willingham M.C., Sui G., Inoue K. (2015). DMP1beta, a splice isoform of the tumour suppressor DMP1 locus, induces proliferation and progression of breast cancer. J. Pathol..

[67] Inoue K., Fry E.A. (2016). Aberrant splicing of the DMP1-ARF-MDM2-p53 pathway in cancer. Int. J. Cancer.

[68] Li K., Zhu Z., Luo J., Fang J., Zhou H., Hu M., Maskey N., Yang G. (2015). Impact of chemokine receptor CXCR3 on tumor-infiltrating lymphocyte recruitment associated with favorable prognosis in advanced gastric cancer. Int. J. Clin. Exp. Pathol..

[69] Zhu G., Yan H.H., Pang Y., Jian J., Achyut B.R., Liang X., Weiss J.M., Wiltrout R.H., Hollander M.C., Yang L. (2015). CXCR3 as a molecular target in breast cancer metastasis: Inhibition of tumor cell migration and promotion of host anti-tumor immunity. Oncotarget.

[70] Berchiche Y.A., Sakmar T.P. (2016). CXC Chemokine Receptor 3 Alternative Splice Variants Selectively Activate Different Signaling Pathways. Mol. Pharmacol..

[71] Van der Feltz C., Anthony K., Brilot A., Pomeranz Krummel D.A. (2012). Architecture of the spliceosome. Biochemistry.

[72] Agrawal A.A., Yu L., Smith P.G., Buonamici S. (2018). Targeting splicing abnormalities in cancer. Curr. Opin. Genet. Dev..

[73] Will C.L., Luhrmann R. (2001). Spliceosomal UsnRNP biogenesis, structure and function. Curr. Opin. Cell Biol..

[74] Graveley B.R. (2000). Sorting out the complexity of SR protein functions. RNA.

[75] Long J.C., Caceres J.F. (2009). The SR protein family of splicing factors: Master regulators of gene expression. Biochem. J..

[76] Martinez-Contreras R., Cloutier P., Shkreta L., Fisette J.F., Revil T., Chabot B. (2007). hnRNP proteins and splicing control. Adv. Exp. Med. Biol..

[77] Zhang J., Manley J.L. (2013). Misregulation of pre-mRNA alternative splicing in cancer. Cancer Discov..

[78] Grosso A.R., Martins S., Carmo-Fonseca M. (2008). The emerging role of splicing factors in cancer. EMBO Rep..

[79] Ghigna C., Giordano S., Shen H., Benvenuto F., Castiglioni F., Comoglio P.M., Green M.R., Riva S., Biamonti G. (2005). Cell motility is controlled by SF2/ASF through alternative splicing of the Ron protooncogene. Mol. Cell.

[80] Karni R., de Stanchina E., Lowe S.W., Sinha R., Mu D., Krainer A.R. (2007). The gene encoding the splicing factor SF2/ASF is a proto-oncogene. Nat. Struct. Mol. Biol..

[81] Kedzierska H., Piekielko-Witkowska A. (2017). Splicing factors of SR and hnRNP families as regulators of apoptosis in cancer. Cancer Lett..

[82] Fischer D.C., Noack K., Runnebaum I.B., Watermann D.O., Kieback D.G., Stamm S., Stickeler E. (2004). Expression of splicing factors in human ovarian cancer. Oncol. Rep..

[83] Xiao R., Sun Y., Ding J.H., Lin S., Rose D.W., Rosenfeld M.G., Fu X.D., Li X. (2007). Splicing regulator SC35 is essential for genomic stability and cell proliferation during mammalian organogenesis. Mol. Cell. Biol..

[84] Sharma S., Liao W., Zhou X., Wong D.T., Lichtenstein A. (2011). Exon 11 skipping of E-cadherin RNA downregulates its expression in head and neck cancer cells. Mol. Cancer Ther..

[85] Jia R., Li C., McCoy J.P., Deng C.X., Zheng Z.M. (2010). SRp20 is a proto-oncogene critical for cell proliferation and tumor induction and maintenance. Int. J. Biol. Sci..

[86] Jensen M.A., Wilkinson J.E., Krainer A.R. (2014). Splicing factor SRSF6 promotes hyperplasia of sensitized skin. Nat. Struct. Mol. Biol..

[87] He X., Ee P.L., Coon J.S., Beck W.T. (2004). Alternative splicing of the multidrug resistance protein 1/ATP binding cassette transporter subfamily gene in ovarian cancer creates functional splice variants and is associated with increased expression of the splicing factors PTB and SRp20. Clin. Cancer Res..

[88] Cohen-Eliav M., Golan-Gerstl R., Siegfried Z., Andersen C.L., Thorsen K., Orntoft T.F., Mu D., Karni R. (2013). The splicing factor SRSF6 is amplified and is an oncoprotein in lung and colon cancers. J. Pathol..

[89] Kurokawa K., Akaike Y., Masuda K., Kuwano Y., Nishida K., Yamagishi N., Kajita K., Tanahashi T., Rokutan K. (2014). Downregulation of serine/arginine-rich splicing factor 3 induces G1 cell cycle arrest and apoptosis in colon cancer cells. Oncogene.

[90] Tang Y., Horikawa I., Ajiro M., Robles A.I., Fujita K., Mondal A.M., Stauffer J.K., Zheng Z.M., Harris C.C. (2013). Downregulation of splicing factor SRSF3 induces p53beta, an alternatively spliced isoform of p53 that promotes cellular senescence. Oncogene.

[91] Dvinge H., Kim E., Abdel-Wahab O., Bradley R.K. (2016). RNA splicing factors as oncoproteins and tumour suppressors. Nat. Rev. Cancer.

[92] David C.J., Chen M., Assanah M., Canoll P., Manley J.L. (2010). HnRNP proteins controlled by c-Myc deregulate pyruvate kinase mRNA splicing in cancer. Nature.

[93] Patry C., Bouchard L., Labrecque P., Gendron D., Lemieux B., Toutant J., Lapointe E., Wellinger R., Chabot B. (2003). Small interfering RNA-mediated reduction in heterogeneous nuclear ribonucleoparticule A1/A2 proteins induces apoptosis in human cancer cells but not in normal mortal cell lines. Cancer Res..

[94] Golan-Gerstl R., Cohen M., Shilo A., Suh S.S., Bakacs A., Coppola L., Karni R. (2011). Splicing factor hnRNP A2/B1 regulates tumor suppressor gene splicing and is an oncogenic driver in glioblastoma. Cancer Res..

[95] Chen M., Zhang J., Manley J.L. (2010). Turning on a fuel switch of cancer: HnRNP proteins regulate alternative splicing of pyruvate kinase mRNA. Cancer Res..

[96] Balasubramani M., Day B.W., Schoen R.E., Getzenberg R.H. (2006). Altered expression and localization of creatine kinase B, heterogeneous nuclear ribonucleoprotein F, and high mobility group box 1 protein in the nuclear matrix associated with colon cancer. Cancer Res..

[97] Jung H., Lee D., Lee J., Park D., Kim Y.J., Park W.Y., Hong D., Park P.J., Lee E. (2015). Intron retention is a widespread mechanism of tumor-suppressor inactivation. Nat. Genet..

[98] Kandoth C., McLellan M.D., Vandin F., Ye K., Niu B., Lu C., Xie M., Zhang Q., McMichael J.F., Wyczalkowski M.A. (2013). Mutational landscape and significance across 12 major cancer types. Nature.

[99] Supek F., Minana B., Valcarcel J., Gabaldon T., Lehner B. (2014). Synonymous mutations frequently act as driver mutations in human cancers. Cell.

[100] Saez B., Walter M.J., Graubert T.A. (2017). Splicing factor gene mutations in hematologic malignancies. Blood.

[101] Larsson C.A., Cote G., Quintas-Cardama A. (2013). The changing mutational landscape of acute myeloid leukemia and myelodysplastic syndrome. Mol. Cancer Res..

[102] Boultwood J., Dolatshad H., Varanasi S.S., Yip B.H., Pellagatti A. (2014). The role of splicing factor mutations in the pathogenesis of the myelodysplastic syndromes. Adv. Biol. Regul..

[103] Malcovati L., Papaemmanuil E., Bowen D.T., Boultwood J., Della Porta M.G., Pascutto C., Travaglino E., Groves M.J., Godfrey A.L., Ambaglio I. (2011). Clinical significance of SF3B1 mutations in myelodysplastic syndromes and myelodysplastic/myeloproliferative neoplasms. Blood.

[104] Thol F., Kade S., Schlarmann C., Loffeld P., Morgan M., Krauter J., Wlodarski M.W., Kolking B., Wichmann M., Gorlich K. (2012). Frequency and prognostic impact of mutations in SRSF2, U2AF1, and ZRSR2 in patients with myelodysplastic syndromes. Blood.

[105] Makishima H., Visconte V., Sakaguchi H., Jankowska A.M., Abu Kar S., Jerez A., Przychodzen B., Bupathi M., Guinta K., Afable M.G. (2012). Mutations in the spliceosome machinery, a novel and ubiquitous pathway in leukemogenesis. Blood.

[106] Papaemmanuil E., Gerstung M., Bullinger L., Gaidzik V.I., Paschka P., Roberts N.D., Potter N.E., Heuser M., Thol F., Bolli N. (2016). Genomic Classification and Prognosis in Acute Myeloid Leukemia. N. Engl. J. Med..

[107] Jiang Y., Zhu Y., Liu Z.J., Ouyang S. (2017). The emerging roles of the DDX41 protein in immunity and diseases. Protein Cell.

[108] Lewinsohn M., Brown A.L., Weinel L.M., Phung C., Rafidi G., Lee M.K., Schreiber A.W., Feng J., Babic M., Chong C.E. (2016). Novel germ line DDX41 mutations define families with a lower age of MDS/AML onset and lymphoid malignancies. Blood.

[109] Zhou J., Chng W.J. (2017). Aberrant RNA splicing and mutations in spliceosome complex in acute myeloid leukemia. Stem Cell Investig..

[110] Daubner G.M., Clery A., Jayne S., Stevenin J., Allain F.H. (2012). A syn-anti conformational difference allows SRSF2 to recognize guanines and cytosines equally well. EMBO J..

[111] Zhang J., Lieu Y.K., Ali A.M., Penson A., Reggio K.S., Rabadan R., Raza A., Mukherjee S., Manley J.L. (2015). Disease-associated mutation in SRSF2 misregulates splicing by altering RNA-binding affinities. Proc. Natl. Acad. Sci. USA.

[112] Kim E., Ilagan J.O., Liang Y., Daubner G.M., Lee S.C., Ramakrishnan A., Li Y., Chung Y.R., Micol J.B., Murphy M.E. (2015). SRSF2 Mutations Contribute to Myelodysplasia by Mutant-Specific Effects on Exon Recognition. Cancer Cell.

[113] Druker B.J., Guilhot F., O’Brien S.G., Gathmann I., Kantarjian H., Gattermann N., Deininger M.W., Silver R.T., Goldman J.M., Stone R.M. (2006). Five-year follow-up of patients receiving imatinib for chronic myeloid leukemia. N. Engl. J. Med..

[114] Hughes T.P., Hochhaus A., Branford S., Muller M.C., Kaeda J.S., Foroni L., Druker B.J., Guilhot F., Larson R.A., O’Brien S.G. (2010). Long-term prognostic significance of early molecular response to imatinib in newly diagnosed chronic myeloid leukemia: An analysis from the International Randomized Study of Interferon and STI571 (IRIS). Blood.

[115] Itonaga H., Tsushima H., Imanishi D., Hata T., Doi Y., Mori S., Sasaki D., Hasegawa H., Matsuo E., Nakashima J. (2014). Molecular analysis of the BCR-ABL1 kinase domain in chronic-phase chronic myelogenous leukemia treated with tyrosine kinase inhibitors in practice: Study by the Nagasaki CML Study Group. Leuk. Res..

[116] Berman E., Jhanwar S., Hedvat C., Arcila M.E., Wahab O.A., Levine R., Maloy M., Ma W., Albitar M. (2016). Resistance to imatinib in patients with chronic myelogenous leukemia and the splice variant BCR-ABL1(35INS). Leuk. Res..

[117] Gaillard J.B., Arnould C., Bravo S., Donadio D., Exbrayat C., Jourdan E., Reboul D., Chiesa J., Lavabre-Bertrand T. (2010). Exon 7 deletion in the bcr-abl gene is frequent in chronic myeloid leukemia patients and is not correlated with resistance against imatinib. Mol. Cancer Ther..

[118] Laudadio J., Deininger M.W., Mauro M.J., Druker B.J., Press R.D. (2008). An intron-derived insertion/truncation mutation in the BCR-ABL kinase domain in chronic myeloid leukemia patients undergoing kinase inhibitor therapy. J. Mol. Diagn..

[119] O’Hare T., Zabriskie M.S., Eide C.A., Agarwal A., Adrian L.T., You H., Corbin A.S., Yang F., Press R.D., Rivera V.M. (2011). The BCR-ABL35INS insertion/truncation mutant is kinase-inactive and does not contribute to tyrosine kinase inhibitor resistance in chronic myeloid leukemia. Blood.

[120] Lee T.S., Ma W., Zhang X., Giles F., Cortes J., Kantarjian H., Albitar M. (2008). BCR-ABL alternative splicing as a common mechanism for imatinib resistance: Evidence from molecular dynamics simulations. Mol. Cancer Ther..

[121] Antonarakis E.S., Lu C., Wang H., Luber B., Nakazawa M., Roeser J.C., Chen Y., Mohammad T.A., Fedor H.L., Lotan T.L. (2014). AR-V7 and resistance to enzalutamide and abiraterone in prostate cancer. N. Engl. J. Med..

[122] Wang B.D., Ceniccola K., Hwang S., Andrawis R., Horvath A., Freedman J.A., Olender J., Knapp S., Ching T., Garmire L. (2017). Alternative splicing promotes tumour aggressiveness and drug resistance in African American prostate cancer. Nat. Commun..

[123] Siegfried Z., Karni R. (2018). The role of alternative splicing in cancer drug resistance. Curr. Opin. Genet. Dev..

[124] Ng K.P., Hillmer A.M., Chuah C.T., Juan W.C., Ko T.K., Teo A.S., Ariyaratne P.N., Takahashi N., Sawada K., Fei Y. (2012). A common BIM deletion polymorphism mediates intrinsic resistance and inferior responses to tyrosine kinase inhibitors in cancer. Nat. Med..

[125] Wang Y., Bernhardy A.J., Cruz C., Krais J.J., Nacson J., Nicolas E., Peri S., van der Gulden H., van der Heijden I., O’Brien S.W. (2016). The BRCA1-Delta11q Alternative Splice Isoform Bypasses Germline Mutations and Promotes Therapeutic Resistance to PARP Inhibition and Cisplatin. Cancer Res..

[126] Poulikakos P.I., Persaud Y., Janakiraman M., Kong X., Ng C., Moriceau G., Shi H., Atefi M., Titz B., Gabay M.T. (2011). RAF inhibitor resistance is mediated by dimerization of aberrantly spliced BRAF(V600E). Nature.

[127] Sotillo E., Barrett D.M., Black K.L., Bagashev A., Oldridge D., Wu G., Sussman R., Lanauze C., Ruella M., Gazzara M.R. (2015). Convergence of Acquired Mutations and Alternative Splicing of CD19 Enables Resistance to CART-19 Immunotherapy. Cancer Discov..

[128] Kuroda J., Puthalakath H., Cragg M.S., Kelly P.N., Bouillet P., Huang D.C., Kimura S., Ottmann O.G., Druker B.J., Villunger A. (2006). Bim and Bad mediate imatinib-induced killing of Bcr/Abl+ leukemic cells, and resistance due to their loss is overcome by a BH3 mimetic. Proc. Natl. Acad. Sci. USA.

[129] Ko T.K., Chin H.S., Chuah C.T., Huang J.W., Ng K.P., Khaw S.L., Huang D.C., Ong S.T. (2016). The BIM deletion polymorphism: A paradigm of a permissive interaction between germline and acquired TKI resistance factors in chronic myeloid leukemia. Oncotarget.

[130] Liu J., Bhadra M., Sinnakannu J.R., Yue W.L., Tan C.W., Rigo F., Ong S.T., Roca X. (2017). Overcoming imatinib resistance conferred by the BIM deletion polymorphism in chronic myeloid leukemia with splice-switching antisense oligonucleotides. Oncotarget.

[131] Moynahan M.E., Cui T.Y., Jasin M. (2001). Homology-directed dna repair, mitomycin-c resistance, and chromosome stability is restored with correction of a Brca1 mutation. Cancer Res..

[132] Scully R., Chen J., Ochs R.L., Keegan K., Hoekstra M., Feunteun J., Livingston D.M. (1997). Dynamic changes of BRCA1 subnuclear location and phosphorylation state are initiated by DNA damage. Cell.

[133] Friedman L.S., Ostermeyer E.A., Szabo C.I., Dowd P., Lynch E.D., Rowell S.E., King M.C. (1994). Confirmation of BRCA1 by analysis of germline mutations linked to breast and ovarian cancer in ten families. Nat. Genet..

[134] Szabo C.I., King M.C. (1995). Inherited breast and ovarian cancer. Hum. Mol. Genet..

[135] Bryant H.E., Schultz N., Thomas H.D., Parker K.M., Flower D., Lopez E., Kyle S., Meuth M., Curtin N.J., Helleday T. (2005). Specific killing of BRCA2-deficient tumours with inhibitors of poly(ADP-ribose) polymerase. Nature.

[136] Farmer H., McCabe N., Lord C.J., Tutt A.N., Johnson D.A., Richardson T.B., Santarosa M., Dillon K.J., Hickson I., Knights C. (2005). Targeting the DNA repair defect in BRCA mutant cells as a therapeutic strategy. Nature.

[137] Liu C., Srihari S., Cao K.A., Chenevix-Trench G., Simpson P.T., Ragan M.A., Khanna K.K. (2014). A fine-scale dissection of the DNA double-strand break repair machinery and its implications for breast cancer therapy. Nucleic Acids Res..

[138] Ledermann J., Harter P., Gourley C., Friedlander M., Vergote I., Rustin G., Scott C.L., Meier W., Shapira-Frommer R., Safra T. (2014). Olaparib maintenance therapy in patients with platinum-sensitive relapsed serous ovarian cancer: A preplanned retrospective analysis of outcomes by BRCA status in a randomised phase 2 trial. Lancet Oncol..

[139] Kim Y., Kim A., Sharip A., Sharip A., Jiang J., Yang Q., Xie Y. (2017). Reverse the Resistance to PARP Inhibitors. Int. J. Biol. Sci..

[140] Thompson D., Easton D., Breast Cancer Linkage Consortium (2002). Variation in BRCA1 cancer risks by mutation position. Cancer Epidemiol. Biomark. Prev..

[141] Risch H.A., McLaughlin J.R., Cole D.E., Rosen B., Bradley L., Fan I., Tang J., Li S., Zhang S., Shaw P.A. (2006). Population BRCA1 and BRCA2 mutation frequencies and cancer penetrances: A kin-cohort study in Ontario, Canada. J. Natl. Cancer Inst..

[142] Risch H.A., McLaughlin J.R., Cole D.E., Rosen B., Bradley L., Kwan E., Jack E., Vesprini D.J., Kuperstein G., Abrahamson J.L. (2001). Prevalence and penetrance of germline BRCA1 and BRCA2 mutations in a population series of 649 women with ovarian cancer. Am. J. Hum. Genet..

[143] Meyer S., Stevens A., Paredes R., Schneider M., Walker M.J., Williamson A.J.K., Gonzalez-Sanchez M.B., Smetsers S., Dalal V., Teng H.Y. (2017). Acquired cross-linker resistance associated with a novel spliced BRCA2 protein variant for molecular phenotyping of BRCA2 disruption. Cell Death Dis..

[144] Surget S., Khoury M.P., Bourdon J.C. (2013). Uncovering the role of p53 splice variants in human malignancy: A clinical perspective. Onco-Targets Ther..

[145] Bourdon J.C., Khoury M.P., Diot A., Baker L., Fernandes K., Aoubala M., Quinlan P., Purdie C.A., Jordan L.B., Prats A.C. (2011). p53 mutant breast cancer patients expressing p53gamma have as good a prognosis as wild-type p53 breast cancer patients. Breast Cancer Res..

[146] Avery-Kiejda K.A., Zhang X.D., Adams L.J., Scott R.J., Vojtesek B., Lane D.P., Hersey P. (2008). Small molecular weight variants of p53 are expressed in human melanoma cells and are induced by the DNA-damaging agent cisplatin. Clin. Cancer Res..

[147] Song W., Huo S.W., Lu J.J., Liu Z., Fang X.L., Jin X.B., Yuan M.Z. (2009). Expression of p53 isoforms in renal cell carcinoma. Chin. Med. J..

[148] Davies H., Bignell G.R., Cox C., Stephens P., Edkins S., Clegg S., Teague J., Woffendin H., Garnett M.J., Bottomley W. (2002). Mutations of the BRAF gene in human cancer. Nature.

[149] Weber C.K., Slupsky J.R., Kalmes H.A., Rapp U.R. (2001). Active Ras induces heterodimerization of cRaf and BRaf. Cancer Res..

[150] Rushworth L.K., Hindley A.D., O’Neill E., Kolch W. (2006). Regulation and role of Raf-1/B-Raf heterodimerization. Mol. Cell. Biol..

[151] Wellbrock C., Karasarides M., Marais R. (2004). The RAF proteins take centre stage. Nat. Rev. Mol. Cell. Biol..

[152] Flaherty K.T., Puzanov I., Kim K.B., Ribas A., McArthur G.A., Sosman J.A., O’Dwyer P.J., Lee R.J., Grippo J.F., Nolop K. (2010). Inhibition of mutated, activated BRAF in metastatic melanoma. N. Engl. J. Med..

[153] Shi H., Hugo W., Kong X., Hong A., Koya R.C., Moriceau G., Chodon T., Guo R., Johnson D.B., Dahlman K.B. (2014). Acquired resistance and clonal evolution in melanoma during BRAF inhibitor therapy. Cancer Discov..

[154] Salton M., Kasprzak W.K., Voss T., Shapiro B.A., Poulikakos P.I., Misteli T. (2015). Inhibition of vemurafenib-resistant melanoma by interference with pre-mRNA splicing. Nat. Commun..

[155] Kalos M., Levine B.L., Porter D.L., Katz S., Grupp S.A., Bagg A., June C.H. (2011). T cells with chimeric antigen receptors have potent antitumor effects and can establish memory in patients with advanced leukemia. Sci. Transl. Med..

[156] Porter D.L., Levine B.L., Kalos M., Bagg A., June C.H. (2011). Chimeric antigen receptor-modified T cells in chronic lymphoid leukemia. N. Engl. J. Med..

[157] Wang Y., Brooks S.R., Li X., Anzelon A.N., Rickert R.C., Carter R.H. (2002). The physiologic role of CD19 cytoplasmic tyrosines. Immunity.

[158] Chung E.Y., Psathas J.N., Yu D., Li Y., Weiss M.J., Thomas-Tikhonenko A. (2012). CD19 is a major B cell receptor-independent activator of MYC-driven B-lymphomagenesis. J. Clin. Investig..

[159] Rickert R.C., Rajewsky K., Roes J. (1995). Impairment of T-cell-dependent B-cell responses and B-1 cell development in CD19-deficient mice. Nature.

[160] Poe J.C., Minard-Colin V., Kountikov E.I., Haas K.M., Tedder T.F. (2012). A c-Myc and surface CD19 signaling amplification loop promotes B cell lymphoma development and progression in mice. J. Immunol..

[161] Wang Z., Wu Z., Liu Y., Han W. (2017). New development in CAR-T cell therapy. J. Hematol. Oncol..

[162] Maude S.L., Frey N., Shaw P.A., Aplenc R., Barrett D.M., Bunin N.J., Chew A., Gonzalez V.E., Zheng Z., Lacey S.F. (2014). Chimeric antigen receptor T cells for sustained remissions in leukemia. N. Engl. J. Med..

[163] Topp M.S., Gokbuget N., Zugmaier G., Klappers P., Stelljes M., Neumann S., Viardot A., Marks R., Diedrich H., Faul C. (2014). Phase II trial of the anti-CD19 bispecific T cell-engager blinatumomab shows hematologic and molecular remissions in patients with relapsed or refractory B-precursor acute lymphoblastic leukemia. J. Clin. Oncol..

[164] Dehm S.M., Schmidt L.J., Heemers H.V., Vessella R.L., Tindall D.J. (2008). Splicing of a novel androgen receptor exon generates a constitutively active androgen receptor that mediates prostate cancer therapy resistance. Cancer Res..

[165] Hu R., Dunn T.A., Wei S., Isharwal S., Veltri R.W., Humphreys E., Han M., Partin A.W., Vessella R.L., Isaacs W.B. (2009). Ligand-independent androgen receptor variants derived from splicing of cryptic exons signify hormone-refractory prostate cancer. Cancer Res..

[166] Wang B.D., Yang Q., Ceniccola K., Bianco F., Andrawis R., Jarrett T., Frazier H., Patierno S.R., Lee N.H. (2013). Androgen receptor-target genes in African American prostate cancer disparities. Prostate Cancer.

[167] Hu R., Lu C., Mostaghel E.A., Yegnasubramanian S., Gurel M., Tannahill C., Edwards J., Isaacs W.B., Nelson P.S., Bluemn E. (2012). Distinct transcriptional programs mediated by the ligand-dependent full-length androgen receptor and its splice variants in castration-resistant prostate cancer. Cancer Res..

[168] Mashima T., Okabe S., Seimiya H. (2010). Pharmacological targeting of constitutively active truncated androgen receptor by nigericin and suppression of hormone-refractory prostate cancer cell growth. Mol. Pharmacol..

[169] Tummala R., Lou W., Gao A.C., Nadiminty N. (2017). Quercetin Targets hnRNPA1 to Overcome Enzalutamide Resistance in Prostate Cancer Cells. Mol. Cancer Ther..

[170] Nadiminty N., Tummala R., Liu C., Lou W., Evans C.P., Gao A.C. (2015). NF-kappaB2/p52:c-Myc:hnRNPA1 Pathway Regulates Expression of Androgen Receptor Splice Variants and Enzalutamide Sensitivity in Prostate Cancer. Mol. Cancer Ther..

[171] Ko C.C., Chen Y.J., Chen C.T., Liu Y.C., Cheng F.C., Hsu K.C., Chow L.P. (2014). Chemical proteomics identifies heterogeneous nuclear ribonucleoprotein (hnRNP) A1 as the molecular target of quercetin in its anti-cancer effects in PC-3 cells. J. Biol. Chem..

[172] Marcinkiewicz C., Galasinski W., Gindzienski A. (1995). EF-1 alpha is a target site for an inhibitory effect of quercetin in the peptide elongation process. Acta Biochim. Pol..

[173] Thomas C., Gustafsson J.A. (2015). Estrogen receptor mutations and functional consequences for breast cancer. Trends Endocrinol. MeTable.

[174] Inoue K., Fry E.A. (2015). Aberrant Splicing of Estrogen Receptor, HER2, and CD44 Genes in Breast Cancer. Genet. Epigenet..

[175] Barone I., Brusco L., Fuqua S.A. (2010). Estrogen receptor mutations and changes in downstream gene expression and signaling. Clin. Cancer Res..

[176] Su X., Xu X., Li G., Lin B., Cao J., Teng L. (2014). ER-α36: A novel biomarker and potential therapeutic target in breast cancer. Onco-Targets Ther..

[177] Zou Y., Ding L., Coleman M., Wang Z. (2009). Estrogen receptor-α (ER-α) suppresses expression of its variant ER-α 36. FEBS Lett..

[178] Shi L., Dong B., Li Z., Lu Y., Ouyang T., Li J., Wang T., Fan Z., Fan T., Lin B. (2009). Expression of ER-α36, a novel variant of estrogen receptor α, and resistance to tamoxifen treatment in breast cancer. J. Clin. Oncol..

[179] Wang Z.Y., Yin L. (2015). Estrogen receptor alpha-36 (ER-α36): A new player in human breast cancer. Mol. Cell. Endocrinol..

[180] Wang Q., Jiang J., Ying G., Xie X.Q., Zhang X., Xu W., Zhang X., Song E., Bu H., Ping Y.F. (2018). Tamoxifen enhances stemness and promotes metastasis of ERα36(+) breast cancer by upregulating ALDH1A1 in cancer cells. Cell Res..

[181] Zhang X., Wang Z.Y. (2013). Estrogen receptor-alpha variant, ER-α36, is involved in tamoxifen resistance and estrogen hypersensitivity. Endocrinology.

[182] Fruman D.A., Rommel C. (2014). PI3K and cancer: Lessons, challenges and opportunities. Nat. Rev. Drug Discov..

[183] Berndt A., Miller S., Williams O., Le D.D., Houseman B.T., Pacold J.I., Gorrec F., Hon W.C., Liu Y., Rommel C. (2010). The p110delta structure: Mechanisms for selectivity and potency of new PI(3)K inhibitors. Nat. Chem. Biol..

[184] Yang Q., Modi P., Newcomb T., Queva C., Gandhi V. (2015). Idelalisib: First-in-Class PI3K Delta Inhibitor for the Treatment of Chronic Lymphocytic Leukemia, Small Lymphocytic Leukemia, and Follicular Lymphoma. Clin. Cancer Res..

[185] Gopal A.K., Kahl B.S., de Vos S., Wagner-Johnston N.D., Schuster S.J., Jurczak W.J., Flinn I.W., Flowers C.R., Martin P., Viardot A. (2014). PI3Kdelta inhibition by idelalisib in patients with relapsed indolent lymphoma. N. Engl. J. Med..

[186] Brown J.R., Byrd J.C., Coutre S.E., Benson D.M., Flinn I.W., Wagner-Johnston N.D., Spurgeon S.E., Kahl B.S., Bello C., Webb H.K. (2014). Idelalisib, an inhibitor of phosphatidylinositol 3-kinase p110delta, for relapsed/refractory chronic lymphocytic leukemia. Blood.

[187] Shah A., Mangaonkar A. (2015). Idelalisib: A Novel PI3Kdelta Inhibitor for Chronic Lymphocytic Leukemia. Ann. Pharmacother..

[188] Lee S.C., Abdel-Wahab O. (2016). Therapeutic targeting of splicing in cancer. Nat. Med..

[189] Antonopoulou E., Ladomery M. (2018). Targeting Splicing in Prostate Cancer. Int. J. Mol. Sci..

[190] Salton M., Misteli T. (2016). Small Molecule Modulators of Pre-mRNA Splicing in Cancer Therapy. Trends Mol. Med..

[191] Seiler M., Yoshimi A., Darman R., Chan B., Keaney G., Thomas M., Agrawal A.A., Caleb B., Csibi A., Sean E. (2018). H3B-8800, an orally available small-molecule splicing modulator, induces lethality in spliceosome-mutant cancers. Nat. Med..

[192] Fukuhara T., Hosoya T., Shimizu S., Sumi K., Oshiro T., Yoshinaka Y., Suzuki M., Yamamoto N., Herzenberg L.A., Herzenberg L.A. (2006). Utilization of host SR protein kinases and RNA-splicing machinery during viral replication. Proc. Natl. Acad. Sci. USA.

[193] Amin E.M., Oltean S., Hua J., Gammons M.V., Hamdollah-Zadeh M., Welsh G.I., Cheung M.K., Ni L., Kase S., Rennel E.S. (2011). WT1 mutants reveal SRPK1 to be a downstream angiogenesis target by altering VEGF splicing. Cancer Cell.

[194] Siqueira R.P., Barbosa Ede A., Poleto M.D., Righetto G.L., Seraphim T.V., Salgado R.L., Ferreira J.G., Barros M.V., de Oliveira L.L., Laranjeira A.B. (2015). Potential Antileukemia Effect and Structural Analyses of SRPK Inhibition by *N*-(2-(Piperidin-1-yl)-5-(Trifluoromethyl)Phenyl)Isonicotinamide (SRPIN340). PLoS ONE.

[195] Mavrou A., Brakspear K., Hamdollah-Zadeh M., Damodaran G., Babaei-Jadidi R., Oxley J., Gillatt D.A., Ladomery M.R., Harper S.J., Bates D.O. (2015). Serine-arginine protein kinase 1 (SRPK1) inhibition as a potential novel targeted therapeutic strategy in prostate cancer. Oncogene.

[196] Araki S., Dairiki R., Nakayama Y., Murai A., Miyashita R., Iwatani M., Nomura T., Nakanishi O. (2015). Inhibitors of CLK protein kinases suppress cell growth and induce apoptosis by modulating pre-mRNA splicing. PLoS ONE.

[197] Dewaele M., Tabaglio T., Willekens K., Bezzi M., Teo S.X., Low D.H., Koh C.M., Rambow F., Fiers M., Rogiers A. (2016). Antisense oligonucleotide-mediated *MDM4* exon 6 skipping impairs tumor growth. J. Clin. Investig..

[198] Hong D., Kurzrock R., Kim Y., Woessner R., Younes A., Nemunaitis J., Fowler N., Zhou T., Schmidt J., Jo M. (2015). AZD9150, a next-generation antisense oligonucleotide inhibitor of STAT3 with early evidence of clinical activity in lymphoma and lung cancer. Sci. Transl. Med..

[199] Ross S.J., Revenko A.S., Hanson L.L., Ellston R., Staniszewska A., Whalley N., Pandey S.K., Revill M., Rooney C., Buckett L.K. (2017). Targeting KRAS-dependent tumors with AZD4785, a high-affinity therapeutic antisense oligonucleotide inhibitor of KRAS. Sci. Transl. Med..

[200] Bauman J.A., Li S.D., Yang A., Huang L., Kole R. (2010). Anti-tumor activity of splice-switching oligonucleotides. Nucleic Acids Res..

[201] Galletti G., Matov A., Beltran H., Fontugne J., Miguel Mosquera J., Cheung C., MacDonald T.Y., Sung M., O’Toole S., Kench J.G. (2014). ERG induces taxane resistance in castration-resistant prostate cancer. Nat. Commun..

[202] Hagen R.M., Adamo P., Karamat S., Oxley J., Aning J.J., Gillatt D., Persad R., Ladomery M.R., Rhodes A. (2014). Quantitative analysis of ERG expression and its splice isoforms in formalin-fixed, paraffin-embedded prostate cancer samples: Association with seminal vesicle invasion and biochemical recurrence. Am. J. Clin. Pathol..

[203] Hammond S.M., Wood M.J. (2011). Genetic therapies for RNA mis-splicing diseases. Trends Genet..

[204] Takeda J., Suzuki Y., Sakate R., Sato Y., Seki M., Irie T., Takeuchi N., Ueda T., Nakao M., Sugano S. (2008). Low conservation and species-specific evolution of alternative splicing in humans and mice: Comparative genomics analysis using well-annotated full-length cDNAs. Nucleic Acids Res..

[205] Moroy T., Heyd F. (2007). The impact of alternative splicing in vivo: Mouse models show the way. RNA.

[206] Li Y., Sun N., Lu Z., Sun S., Huang J., Chen Z., He J. (2017). Prognostic alternative mRNA splicing signature in non-small cell lung cancer. Cancer Lett..

[207] Zhu J., Chen Z., Yong L. (2018). Systematic profiling of alternative splicing signature reveals prognostic predictor for ovarian cancer. Gynecol. Oncol..

[208] Bjorklund S.S., Panda A., Kumar S., Seiler M., Robinson D., Gheeya J., Yao M., Alnaes G.I.G., Toppmeyer D., Riis M. (2017). Widespread alternative exon usage in clinically distinct subtypes of Invasive Ductal Carcinoma. Sci. Rep..

[209] Robertson A.G., Shih J., Yau C., Gibb E.A., Oba J., Mungall K.L., Hess J.M., Uzunangelov V., Walter V., Danilova L. (2017). Integrative Analysis Identifies Four Molecular and Clinical Subsets in Uveal Melanoma. Cancer Cell.

[210] Marcelino Meliso F., Hubert C.G., Favoretto Galante P.A., Penalva L.O. (2017). RNA processing as an alternative route to attack glioblastoma. Hum. Genet..

[211] Kahles A., Lehmann K.V., Toussaint N.C., Huser M., Stark S.G., Sachsenberg T., Stegle O., Kohlbacher O., Sander C., The Cancer Genome Atlas Research Network (2018). Comprehensive Analysis of Alternative Splicing Across Tumors from 8705 Patients. Cancer Cell.

